# Duplications involving the long range *HMX1* enhancer are associated with human isolated bilateral concha-type microtia

**DOI:** 10.1186/s12967-020-02409-6

**Published:** 2020-06-17

**Authors:** Nuo Si, Xiaolu Meng, Xiaosheng Lu, Zhe Liu, Zhan Qi, Lianqing Wang, Chuan Li, Meirong Yang, Ye Zhang, Changchen Wang, Peipei Guo, Lingdong Zhu, Lei Liu, Zhengyong Li, Zhenyu Zhang, Zhen Cai, Bo Pan, Haiyue Jiang, Xue Zhang

**Affiliations:** 1 Plastic Surgery Hospital, Chinese Academy of Medical Sciences and Peking Union Medical College, Beijing, China; 2 Institute of Basic Medical Sciences Chinese Academy of Medical Sciences, School of Basic Medicine Peking, Union Medical College, Beijing, China; 3 Department of Plastic Surgery, Affiliated Hospital of Weifang Medical University, Beijing, China; 4 Laboratory of Clinical Genetics, Peking Union Medical College Hospital, Chinese Academy of Medical Sciences & Peking Union Medical College, Beijing, China; 5 Department of Plastic Surgery, Qilu Children’s Hospital of Shandong University, Jinan, China; 6 Department of Burns and Plastic Surgery, Second Hospital of Shandong University, Beijing, China; 7 Department of Plastic and Burn Surgery, West China School of Medicine, West China Hospital, Sichuan University, Sichuan, China; 8 Department of Plastic Surgery, Sichuan Academy of Medical Sciences & Sichuan Provincial People’s Hospital, Sichuan, China

**Keywords:** Microtia, Duplication, *HMX1*, Long range enhancer, Conserved non-coding elements

## Abstract

**Background:**

Microtia is a congenital anomaly of ear that ranges in severity from mild structural abnormalities to complete absence of the outer ears. Concha-type microtia is considered to be a mild form. The H6 family homeobox 1 transcription factor gene (*HMX1*) plays an important role in craniofacial structures development. Copy number variations (CNVs) of a downstream evolutionarily conserved enhancer region (ECR) of *Hmx1* associated with ear and eye abnormalities have been reported in different animals, but not yet in human. To date, no genetic defects responsible for isolated human microtia has been reported except for mutations in *HOXA2*. Here we recruited five Chinese families with isolated bilateral concha-type microtia, and attempt to identify the underlying genetic causes.

**Methods:**

Single Nucleotide polymorphism (SNP) array was performed to map the disease locus and detect CNVs on a genome scale primarily in the largest family (F1). Whole genome sequencing was performed to screen all SNVs and CNVs in the candidate disease locus. Array comparative genomic hybridization (aCGH) was then performed to detect CNVs in the other four families, F2-F5. Quantitative real-time polymerase chain reaction (qPCR) was used to validate and determine the extent of identified CNVs containing *HMX1*-ECR region. Precise breakpoints in F1 and F2 were identified by gap-PCR and sanger sequencing. Dual-luciferase assays were used to detect the enhancer function. qPCR assays were also used to detect *HMX1*-ECR CNVs in 61 patients with other types mictrotia.

**Results:**

Linkage and haplotype analysis in F1 mapped the disease locus to a 1.9 Mb interval on 4p16.1 containing *HMX1* and its downstream ECR region. Whole genome sequencing detected no potential pathogenic SNVs in coding regions of *HMX1* or other genes within the candidate disease locus, but it detected a 94.6 Kb duplication in an intergenic region between *HMX1* and *CPZ*. aCGH and qPCRs also revealed co-segregated duplications in intergenic region downstream of *HMX1* in the other four families. The 21.8 Kb minimal overlapping region encompassing the core sequences consensus with mouse ECR of Hmx1. Luciferase assays confirmed the enhancer function in human sequences, and proved that HOXA2 could increase its enhancer activity. No CNVs were detected in *HMX1*-ECR regions in 61 patients with other type of microtia.

**Conclusion:**

Duplications involving long range *HMX1* enhancers are associated with human isolated bilateral concha-type microtia. We add to evidences in human that copy number variations in *HMX1*-ECR associates with ear malformations, as in other species. This study also provides an additional example of functional conserved non-coding elements (CNEs) in humans.

## Background

Dumbo, the famous Disney cartoon character, is an elephant with oversized ears that enable it to fly. Some real-life animals with abnormal external ears are also named “dumbo” such as the *dumbo* mouse and *dumbo* rat [1, 2]. Almost all mammals have outer ears (pinna) of variable sizes. The main function of the pinna is to collect sound waves and direct them into the ear. In some species, pinna also serve functions such as dissipating heat and signaling mood. In humans, different congenital pinna malformations are observed. Microtia (OMIM 600674) is an external ear developmental malformation characterized by a small, abnormally shaped pinna [3]. It ranges from mild structural abnormalities to complete absence of the ear, affects one or both ears, and occurs as isolated or syndromic birth defects. The concha-type microtia is considered a mild form of microtia, with remnant ear lobule, concha, acoustic meatus, tragus, and incisura intertragica [4]. Because of the variety of severity and forms, it is hard to estimate microtia prevalence—reported data varies from 0.83 to 17.4 in 10,000 live births worldwide [3]. The causes of microtia among most patients are unknown, although some risk factors have been reported, such as gestational exposure to teratogens, maternal diabetes, and higher maternal parity [3].

Genetic studies have made great progress in understanding ear development and function by identifying underlying genetic defects of certain diseases, especially in hearing loss, but less is known about genetic control of external ear morphogenesis. To date, only *HOXA2* mutations have been reported as responsible for isolated bilateral microtia with or without hearing loss in humans [5–7]. Single-gene defects and chromosomal aberrations have also been reported in different microtia-associated syndromes [3, 8]. Nevertheless, efforts in finding coding region mutations in genes responsible for microtia-associated syndromes have failed for isolated microtia. Among these, the H6 family homeobox 1 transcription factor gene (*HMX1*) in 4p16.1 deserves special attention. It plays an important role downstream of embryonic patterning genes in lateral facial mesenchyme differentiation [1]. In human, recessive loss of function mutations in *HMX1* have been associated with oculoauricular syndrome (OAS, OMIM 142992) characterized by malformation of the external ear and eyes [9, 10]. A linkage locus of 10-Mb encompassing 4p16 has been reported in a five-generation Chinese family with isolated bilateral microtia [11].

In the human genome, 98% of sequences are non-coding but harbor many regulatory elements that direct the precise spatial and temporal expression of coding genes. Comparing genomic sequences from diverse vertebrate species has revealed numerous highly conserved non-coding regions near developmental regulatory genes, particularly transcription factors and these regions are considered to have potential regulatory functions [12]. These functional regions are collectively referred to conserved non-coding elements (CNEs). The general features of CNEs were noticed, including their non-random distribution in line with key developmental regulatory target genes across genomes, the distinguished sequence features with AT-rich and runs of identical nucleotides, the overlapping with transcription factor binding sites and known function as developmental enhancers in many cases [12].Human diseases and phenotypic changes have been associated with alterations in CNEs [13–18]. One of the well-characterized example is the *SHH* ZRS enhancer, in which point mutations and copy number variations could result in limb malformation in both human and other species [19–21]. In wild populations of animals, a CNE proximal to the Hmx1 was also noticed and proved to be associated with external ear development [22].Structural variants (SVs) such as deletions, duplications, insertions and inversions can disrupt or rearrange functional genomic elements [23, 24]. The genetic etiology of many diseases such as limb malformation and autism has been proven to relate to rare inherited SVs in coding gene cis-regulatory elements [25–27]. For ear development, evidence in mice implicated an evolutionarily conserved enhancer region (ECR) downstream of *Hmx1* as an important regulatory element driving ear development. Hoxa2, Meis and Pbx can act cooperatively on a 32 bp core sequence within the ECR to regulate *Hmx1* expression [28]. Mutations in *Hmx1* coding region and SVs involving the *Hmx1*-ECR region have been found in animals with dysmorphic external ears, including ‘dumbo’ or ‘misplaced ears’ in mice, ‘dumbo’ in rats, ‘crop ear’ in highland cattle, and ‘short ear’ in Altay sheep (Table 1) [1, 2, 10, 29, 30]. In human genome, ~ 600 bp conserved sequence homologous to mouse *Hmx1*-ECR was also observed. However, whether genetic changes affecting this region are associated with human ear malformations is unknown.

**Table 1:** Genomic changes and dysmorphic outer ear phenotypes across species

Phenotype description	Phenotype/disease entry	Species	Genomic changes	Inheritance	References
Enlarged ear pinnae with a distinctive ventrolateral shift, microphthalmic anomalies	Dumbo (*dmbo*)	Mouse	Nonsense mutation in *Hmx1* exon1	Recessive	Munroe et al. [1]
Laterally-protruding ears and microphthalmic anomalies	Misplaced ears (*mpe*)	Mouse	8 bp deletion in *Hmx1* exon2	Recessive	Munroe et al. [1]
Congenital malformations of the pinna and modest reduction in ocular size	Dumbo (dmbo)	Rat	5777 bp deletion encompassing Hmx1-ECR	Recessive	Quina et al. [2]
Moderately to severely truncated ear	Crop ear	Highland cattle	76 bp Hmx1-ECR duplication	Dominant	Koch et al. [29]
Shorter and thicker ear	Short ear	Altay sheep	76 bp Hmx1-ECR duplication	Dominant	He et al. [30]
Ophthalmic anomalies and external ear abnormalities	Oculoauricular syndrome (OAS)	Human	26 bp deletion in HMX1 coding region	Recessive	Schorder et al. [10]
Bilateral external ear malformation/cup ear	Concha type microtia	Human	Duplications involving HMX1-ECR	Dominant	This study

In the present study, we show that duplications involving *HMX1*-ECR are associated with human isolated bilateral concha-type microtia. A ~ 600 bp human ECR sequence may function as a tissue-specific enhancer regulating *HMX1* expression and response to *HOXA2* in the lateral facial mesenchyme that contributes to outer ear development.

## Materials and methods

### Subjects

**Fig. 1:**
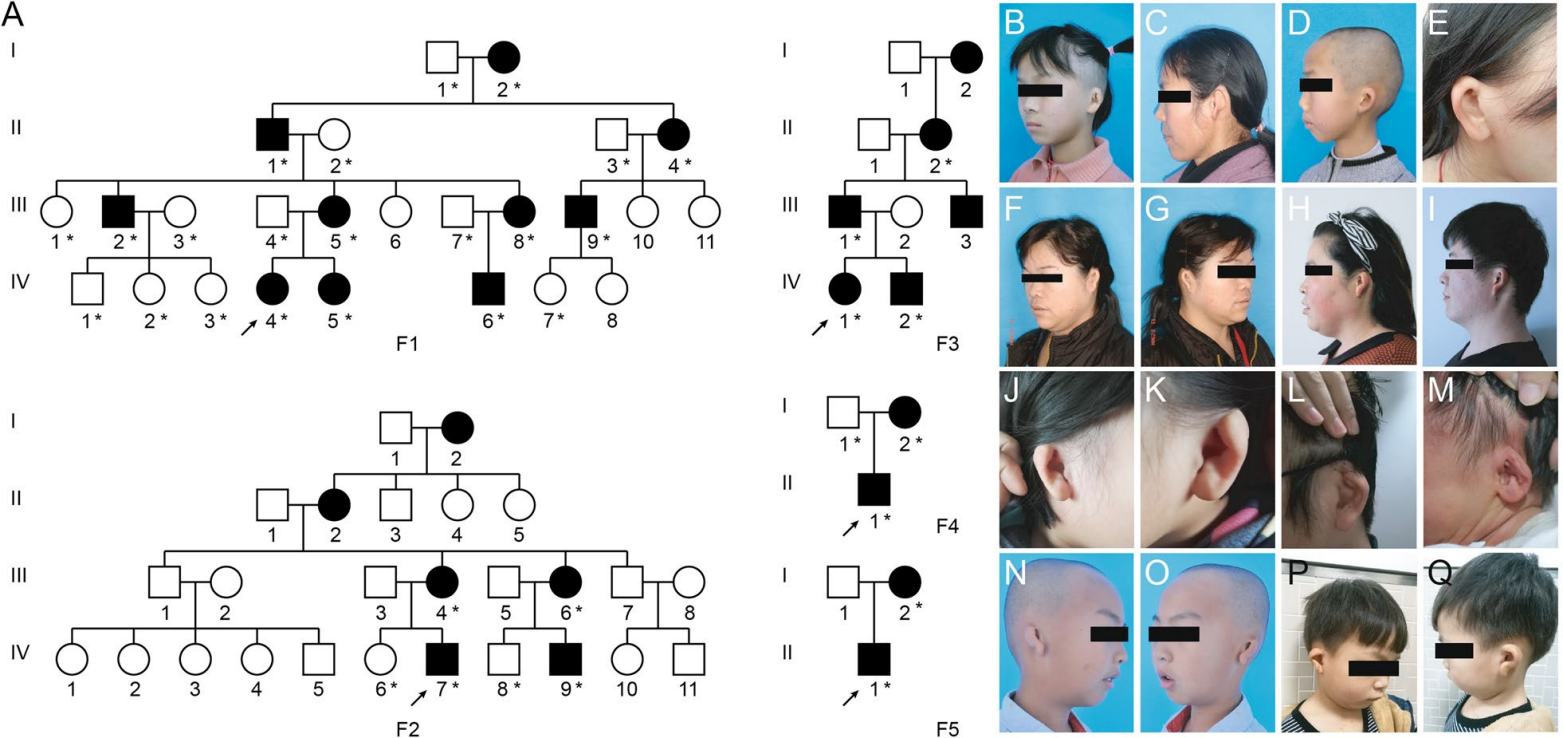
Five families with isolated bilateral Concha-Type Microtia. **a** Pedigree of five families (F1-F5). Individuals with available blood samples are indicated with an asterisk. **b**–**q** Identical pinna phenotypes in five families. All patients have identical bilateral concha-type microtia phenotype, and representative individuals from each family are shown. IV-4 (**b**), III-8 (**c**), IV-6 (**d**), IV-5 (**e**) in F1; III-4 (**f**, **g**), III-6 (**h**), IV-7 (**i**) in F2; IV-1 (**j**, **k**), III-1 (**l**), IV-2 (**m**) in F3; II-1 in F4 (**n**, **o**), II-1 in F5 (**p**, **q**)

Five Han Chinese families with isolated bilateral microtia were included in the present study (Fig. 1). All patients were clinically evaluated, and digital photographs were taken to document ear phenotypes in affected individuals. Family 1 (F1) consisted of 25 individuals including 10 affected individuals with microtia in four generations. Family 2 (F2) and Family 3 (F3) each had six affected individuals in four generations. Family 4 (F4) and Family 5 (F5) are nuclear families with an affected child and affected mother. The ear malformations are consistent within the five families (Fig. 1b–q). We also recruited 53 patients with oculoauricular syndrome, six patients with severe bilateral isolated microtia and two patients with bilateral syndromic microtia. Blood samples from all available family members were collected following informed consent. The study was reviewed and approved by the institutional review board of the Chinese Academy of Medical Sciences.

### Genotyping, whole genome linkage and haplotype analysis

Affymetrix Genome-Wide Human SNP array 5.0 was used to perform whole genome linkage analysis in the four-generation F1 family. Genomic DNA samples from 10 individuals were genotyped following the manufacturer’s instructions. Genotype calling and quality control were performed with the Affymetrix Genotyping Console 2.1 package. Parametric multipoint linkage analysis was performed using MERLIN v.1.1.2 under the assumptions of autosomal-dominant inheritance with 99% penetrance, a disease allele frequency of 0.1%, and equal SNP allele frequency (50%). Genotyping and data analysis were accomplished at the CapitalBio Corporation (Beijing, China). Selected polymorphic micro-satellite markers within candidate disease loci were genotyped. Polymorphic micro-satellite markers and amplification primers are summarized in Additional file 1: Table S1.

### Whole genome sequencing

Ten individuals from F1 (III2, III3, III4, III5, III8, III9, IV1, IV4, IV6, IV7) underwent whole genome sequencing (WGS) using the NEBNext Ultra II DNA Library Prep kit for Illumina (New England Biolabs, Ipswich, MA, USA) and a HiSeq X Ten sequencer (Illumina, San Diego, CA, USA). Reads were aligned to the GRCh37/hg19 human reference sequence using the Burrows-Wheeler Aligner (BWA, v.0.7.8-r455) and variant calling was performed with SAMtools (v.1.0) and annotated using ANNOVAR (v.2015Dec14). Picard (v.1.111) was used to merge BAM files of the same sample and filter out duplicate reads marked. SNP/Indel, CNV, and SV variants were called and classified by SAMtools (v.1.0), Control-FREEC (v.V7.0), and CREST (v.V0.0.1), respectively. WGS was performed and bioinformatic analysis accomplished at the Novogene Corporation (Beijing, China).

### Microarray analysis

Genomic copy number changes at locus 4p16.1 in F2-5 were further tested by the Laboratory of Clinical Genetics of Peking Union Medical College Hospital using a High-Resolution Array CGH analysis (SurePrint G3 Human 1x1M; Agilent Technologies, Santa Clara, CA, USA). One patient from each family was selected to undergo microarray analysis. The experiment and data analysis were performed according to the manufacturer’s instructions. In brief, patient and control DNA were labeled and combined to hybridize to the 60mer oligonucleotide-based microarray. The resulting fluorescent signals were automatically scanned by the Agilent SureScan Microarray Scanner. Agilent CytoGenomics software was then used to extract and translate the signal into log ratios for further analysis of copy number changes.

### Real-time quantitative PCR (qPCR) and Gap-PCR

We performed qPCR to confirm the *HMX1*-ECR duplication and determine the extent of duplications in different families. qPCR primer sequences and amplicon positions are given in Additional file 2: Table S2. qPCR assays were performed using SYBR premix Ex Taq (TaKara Bio., Dalian, China), and reactions were run in a Rotor-gene 6000 real-time rotary analyzer (Qiagen, Hilden, Germany) as previously reported [19]. Data were analyzed by Rotor Gene Q series software (Qiagen, Hilden, Germany). The relative copy number (RCN) of the target sequence was determined by the comparative ΔΔCt method where ΔCt = (mean Ct_Target_) − (mean Ct_Reference_) and ΔΔCt = ΔCt_patient_ − ΔCt_control_. An RCN of ~ 1.5 indicated a heterozygous duplication. For F1 and F2, Gap-PCR was designed according to the extent of duplication implicated by qPCR assays. q20 forward and q3 reverse primers were used for Gap-PCR in F1, while q35 forward and q8 reverse primers were used for Gap-PCR in F2 (Additional file 2: Table S2). Breakpoint junctions were detected by direct Sanger sequencing of Gap-PCR products.

### Dual-luciferase activity assay

hECR and mECR fragments were PCR amplified from genomic DNA and inserted into the pGL4.23 firefly luciferase vector (Promega, Madison, WI, USA) using either a restriction digest strategy or the In-Fusion cloning kit (TaKaRa Bio, Beijing, China). The human HOXA2 cDNA sequence was inserted into the multiple cloning site of the pcDNA3.1(+) vector (Invitrogen, Carlsbad, CA, USA) using *HindIII* and *BamHI*. All plasmids were sequenced to confirm correct fragment insertion. Primers for plasmid construction are summarized in Additional file 3: Table S3. COS-1 cells were plated into 24-well plates 1 day before transfection and grown until 70–90% confluent. For each well, 500 ng luciferase reporter vector was transfected into the cells using Lipofectamin™ 3000 Reagent (Invitrogen, Carlsbad, CA, USA) with or without the pcDNA3.1 expression vector, with 25 ng of the pRL-TK Renilla luciferase vector used as an internal control to normalize transfection efficiency. 24 h post-transfection, cells were harvested and lysed with 100 μl passive lysis buffer (Promega, Madison, WI, USA). The firefly and renilla luciferase activities for each 20 μl cell lysate were measured by the Microplate Luminometer Centro LB 960 (Berthold, Germany). Relative luciferase activity was calculated by the ratio of firefly luciferase activities/renilla luciferase activities as fold change compared to pGL4.23. Assays were conducted as indicated in the dual luciferase reporter assay system manual (Promega, Madison, WI, USA). Normalized luciferase activity fold change (mean ± SD) of three experiments with six duplicates each is reported.

## Results

### Mapping of a susceptibility locus on 4p16.1

Genome-wide linkage analysis in F1 suggested three candidate loci: a 20 Mb interval on 4p16.1, a 25 Mb interval on 4q, and a 2 Mb interval on 5q. Genotyping of selected polymorphic microsatellite markers within candidate regions of 4q and 5q showed no co-segregation status in F1. However, one polymorphic microsatellite marker (CHLC.GATA151E03) on 4p16.1 co-segregated with phenotype in F1. Fine mapping using Affymetrix SNP 5.0 microarray probe-sets refined the critical region to 1.9 Mb between rs4696668 to rs16891285 (chr4:8061832–9954880, hg19) with a HLOD score of 1.8. The interval includes 13 protein-coding genes including *HMX1*, yet we found no potential coding region mutations in these genes by sanger sequencing.

### Identification of the *HMX1*-ECR duplication in five families with isolated bilateral concha-type microtia

**Fig. 2:**
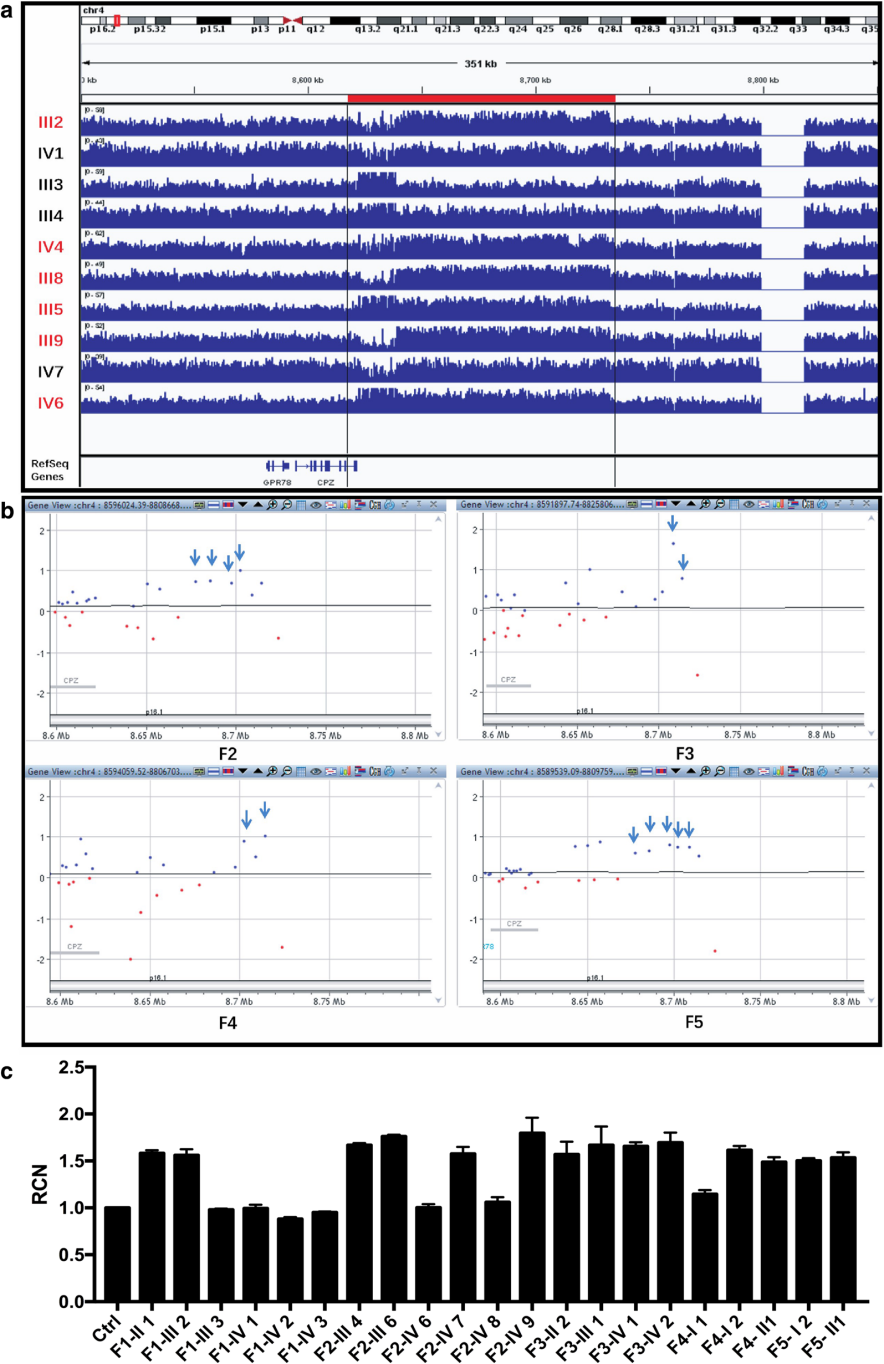
Detected duplications involving the long range *HMX1* Enhancer in five families. **a** Whole genome sequencing indicated duplications in F1. Red bar shows the duplicated region. **b** Duplications detected by array-CGH in F2-F5. Blue arrows show where the probes detected 3 copies. **c** qPCR assays in the *HMX1*-ECR region confirm the duplication and co-segregation status with phenotype in five families

We further performed WGS in 10 members of F1. Consistent with the previous sanger sequencing result, no potential mutations were identified in the coding region. However, WGS implicated a ~ 95 Kb duplication in the critical interval in six patients, but not in two unaffected members or two unrelated members in the family (Fig. 2a). The duplication encompasses a partial intragenic region between *CPZ* and *HMX1*, and involves the ~ 600 bp evolutionarily conserved region downstream of *HMX1* (*HMX1*-ECR). qPCR assays designed within a 600 bp critical region confirmed the duplication and detected full-segregation status in F1 (Fig. 2c). This finding prompted us to detect copy number changes in other families with isolated bilateral concha-type microtia. There are limited probes within the identified duplicated region designed in commercial array CGH systems, decreasing our accuracy and efficiency in CNV detection. Nevertheless, SurePrint G3 Human 1x1M microarray implicated increased copy number in a 46.2 Kb intergenic region (chr4: 8677567–8723767, hg19) between *CPZ* and *HMX1* in four additional families with the identical phenotype same as F1 (Fig. 2b). Duplications were confirmed by qPCR assay in the *HMX1*-ECR region in all four additional families (Fig. 2c).

### Determination of the duplication extent and critical region

**Fig. 3:**
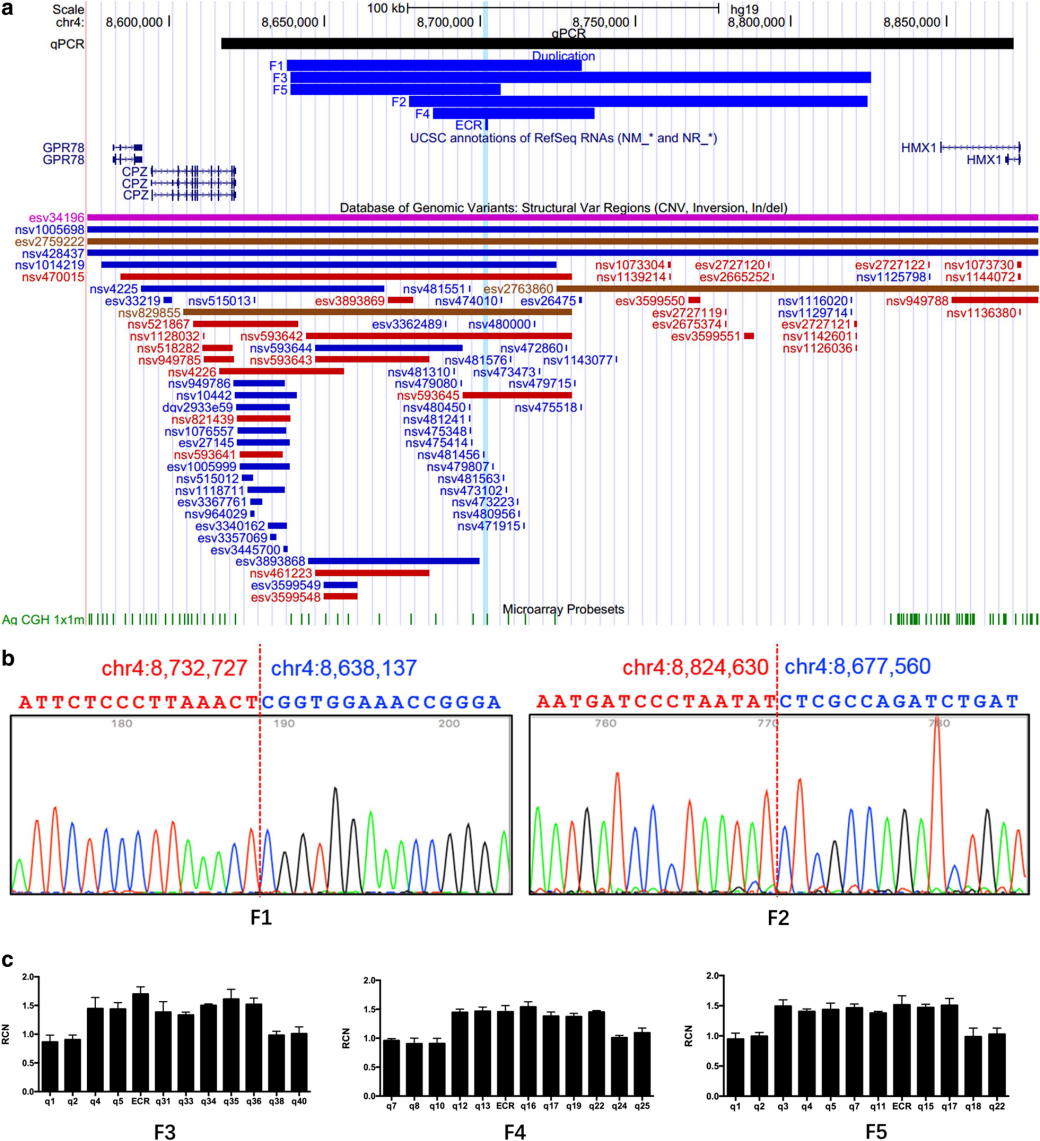
Extent and overlapping regions of duplications in five families. A. Schematic diagram showing detected duplications, 600 bp core hECR sequences, and CNVs in the database of genomic variants. The blue line highlights the ECR region. B. Chromatogram of the breakpoint junctions in F1 and F2. C. A series of qPCR assays detected the extent of duplications in F3, F4 and F5

To determine the size of the duplications in different families, multiple qPCR assays were designed to cover a region of 253 Kb (chr4:8617326–8871246, hg19) encompassing *CPZ*, *HMX1*, and their intergenic region (Additional file 2: Table S2). Extent and overlapping regions of duplications in five families were detected (Fig. 3). We performed qPCR assays on one affected individual per family to determine the extent of duplication in each family and identified duplications of 94.6 Kb, 147 Kb, 185–213 Kb, 49.8–55.9 Kb, and 67.4–104 Kb in F1, F2, F3, F4 and F5, respectively (Fig. 3a). We detected the precise duplicated segment and breakpoints by gap-PCR and sanger sequencing in F1 (chr4:8638135–8732725, hg19) and F2 (chr4: 8677560–8,824,629, hg19) (Fig. 3b). In F3, F4 and F5, multiple qPCR assays detected the boundary regions harboring the breakpoints (Fig. 3c). All identified duplications contained a 21.8 Kb overlapping region (chr4:8,684,896–8,706,719, hg19) harboring the *HMX1*-ECR.

### A 600 bp sequence within the duplicated region shows enhancer activity increased by HOXA2

**Fig. 4:**
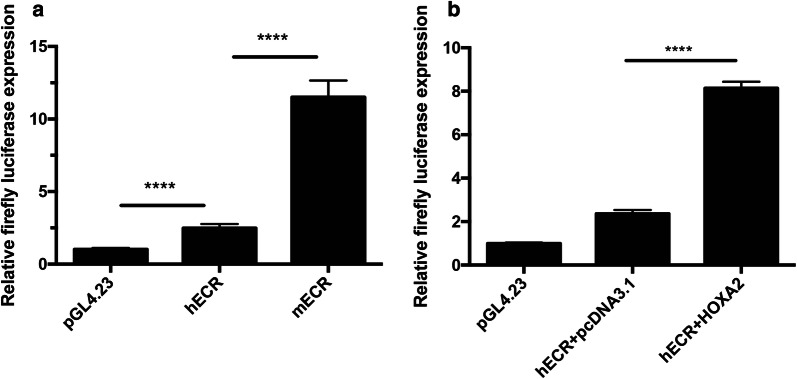
Human ECR within the duplicated region shows an enhancer activity increased by HOXA2. **a** hECR showed increased luciferase activity. **b** hECR is responsive to HOXA2. ****p < *0.0001*

A 594 bp *Hmx1*-ECR region has been demonstrated to be a specific enhancer determining endogenous Hmx1 lateral facial expression patterns in mouse [28]. Thus, a 600 bp human sequence (hECR) in the identified duplicated region homologous to the 594 bp mouse sequence (mECR) was tested for enhancer function by dual luciferase assay (Fig. 4). As a result, constructs containing hECR showed increased luciferase activity compared to the empty group (replicate = 3, *p *< 0.0001), suggesting hECR enhancer activity (Fig. 4a). However, the induced luciferase activity was significantly lower in the hECR than in the mECR group, which is regulated by the Hox-Pbx-Meis complex (Fig. 4a). *HOXA2* mutations were reported in patients with isolated bilateral microtia without hearing loss. In the luciferase assay, co-transfection with human *HOXA2* expression vectors led to an 8.14-fold increase in enhancer activation, indicating that the hECR is responsive to *HOXA2* (Fig. 4b).

### Detection of *HMX1*-ECR CNVs in patients with other types of microtia

To determine whether *HMX1*-ECR CNVs associate with other ear malformations, we performed qPCR assays in the 600 bp *HMX1*-ECR in 53 patients with unilateral lobule-type microtia, six patients with isolated bilateral lobule-type microtia, one patient with bilateral concha-type microtia with preauricular sinus, and one patient with bilateral concha-type microtia with atrial septal defect. No duplications or deletions were detected in these microtia cases. In health population, some duplications involving the *HMX1*-ECR region were documented in the database of genomic variants (DGV) [31], but they were relatively large in size and involved other nearby genes (at least *CPZ*) at the same time. There were also some duplications only involving the *CPZ* and *HMX1* intergenic region, but they did not contain the *HMX1*-ECR. While deletions only involving the intragenic region between *CPZ* and *HMX1*, and containing *HMX1*-ECR were also documented in the DGV database (Fig. 3a).

## Discussion

Microtia is phenotypically and etiologically heterogeneous. Little is known about the genetic background underlying microtia. Among candidate loci for microtia, Chromosome 4p16 deserves special attention. A partial deletion from the short arm of chromosome 4 (4p deletion) results in Wolf-Hirschhorn syndrome (WHS, OMIM#194190) featuring a distinct craniofacial phenotype and intellectual disability [32]. WHS patients with pure and translocated forms of monosomy 4p16.1 → pter (M4p16.1) have different types of external ear malformation such as poorly rolled descending helix edge, short ear lobes, or deep or long concha [33]. By studying 72 oculoauriculovertebral spectrum (OAVS) patients with highly heterogeneous phenotypes involving ears, eyes, face, neck and other organs, Bragagnolo et al. observed recurrent chromosomal imbalances predominantly in chromosome 4 in four patients [34]. Balikova et al. reported on a large family with autosomal-dominantly inherited microtia, eye coloboma, and imperforation of the nasolacrimal duct, and found the phenotype linked to a cytogenetically visible alteration at 4p16 consisting of five copies of a copy-number-variable region [35]. Li et al. reported a 10 Mb susceptibility locus for isolated bilateral microtia on 4p15.32–4p16.2 in a 5-generation Chinese family [11].

*HMX1* harbored in 4p16.1, also known as *NKX5*-*3,* is an important transcription factor in craniofacial structure development, especially in eye and ear. Expression of *Hmx1* was observed in the external ear, lens, and retina of mice as early as E13.5. In humans, *HMX1* expression was observed in the optic vesicle in the 5–6-week embryonic period and in the developing pinna and auricular mesenchymatous cells at the 20-week fetus period [10]. The different expression patterns of *HMX1* in ear and eye development suggest that there may be different regulatory elements determining strict spatial–temporal expression. Meanwhile, homozygous mutation in the human *HMX1* gene leads to abrogation of gene function causing oculoauricular syndrome (OAS, OMIM #612109) affecting both the eye and external ear [9, 10, 36]. Thus, isolated microtia and syndromic microtia without eye affects are unlikely to be caused by mutations in the *HMX1* coding region. Accordingly, we found no potential *HMX1* coding region mutations in 120 OAVS patients by whole exome sequencing (unpublished data).

Conserved non-coding elements (CNEs) are sequences outside of protein coding regions highly conserved across diverse vertebrate species [37]. They may act as cis-regulatory modules (CRMs) that interact with nearby genes to determine tissue-specific gene expression, and they are enriched near transcription factor genes expressed during embryogenesis, suggesting a possible role in regulating the expression of essential developmental genes [38, 39]. CNEs are required for normal development, and mutations in CNEs have been established as causal for human diseases and subtle phenotypic changes that likely lead to decreased fitness over evolutionary time [12]. Dickel et al. created knock out mice with individual or pairwise deletion of four CNEs near *ARX*, the essential neuronal transcription factor [40]. These knockout mice showed substantial alterations of neuron populations and structural brain defects that potentially detrimental in the wild, although they were viable and fertile in laboratory conditions. *Rosin* et al. showed that *Hmx1* has such a CNE that functions as a strong and highly dynamic lateral facial enhancer [28]. The CNE is a ~ 600 bp evolutionarily conserved region (ECR) with a 32 bp core sequence containing consensus binding sites for Hoxa2, Pbx, and Meis, and it has tissue-specific enhancer function in the craniofacial mesenchyme which contributes to the pinna. Genomic structural variations disrupting the ECR enhancer role associate with loss of Hmx1 expression specifically in the first and second branchial arch (BA1 and BA2) mesenchyme, leading to dysmorphic outer ears across species (Table 1). Genomic findings in human patients with isolated bilateral concha-type microtia reinforce the enhancer role of *HMX1*-ECR in conserved pinna developmental processes. We also noticed that hECR has weaker enhancer activity compared to mECR via luciferase assay. However, it remains unclear whether the difference in the relative size of the pinna between human and mice is related to the level of enhancer activity.

The core sequence of hECR is highly homologous to mECR including the consensus binding sites of HOXA2, PBX and MEIS [22, 28]. In dual luciferase assays, co-transfection of HOXA2 and hECR resulted in increased expression level, suggesting that the hECR may also be regulated by the HOX-PBX-MEIS complex. HOX, PBX and MEIS are all homeobox proteins involved in transcriptional regulation by forming heterodimers and are essential contributors to developmental programs. Genes encoding this homeoprotein complex associate with congenital anomalies with craniofacial phenotypes. *HOXA2* is the only reported gene responsible for isolated microtia to date. Patients with homozygous mutations in HOXA2 display more severe microtia than hECR duplicated carriers, presenting middle ear deformities and hearing loss [7]. *PBX1* mutations lead to congenital kidney and urinary tract anomalies with or without hearing loss, abnormal ears, or developmental delay [41]. MEIS2 mutations associate with cleft palate, cardiac defects, and mental retardation [42, 43]. These findings suggest that *HOXA2, PBX1,* and *MEIS2* act early in patterning of the branchial arch region and transactivate *HMX1* by binding to hECR. Therefore, we speculate that any genetic changes affecting hECR regulation by the HOX-PBX-MEIS complex may lead to developmental defects involving ears and eyes.

Regulatory elements and their target gene clusters often exist in the same local chromatin interaction regions, called topologically associated domains (TADs), to ensure that the regulatory elements are specific to their target genes rather than other nearby genes [44]. Boundaries between TADs are required and provide an insulator function to prohibit interference between opposing activities of neighboring domains [44, 45]. We used the 3D Genome Browser (http://promoter.bx.psu.edu/hi-c/) to visualize the chromatin interaction surrounding *HMX1*. According to Hi-C profile data from human embryonic stem cells, *HMX1* and *CPZ* are in two different TADs, while *hECR* sequences appear in the same TAD with *HMX1* but not with *CPZ* (Additional file 4: Figure S1). The detected duplications in isolated bilateral concha-type microtia patients are in the same TAD with *HMX1,* and do not interrupt the TAD boundary. They contain hECR but not the HMX1 gene. Therefore, these duplications may result in overexpression of *HMX1* by increasing the number of local enhancers but not the coding gene. Meanwhile, the copy number variation (nsv1014219) in the DGV database detected in normal population involves the hECR, the CPZ gene, and the boundary between two TADs. Therefore, due to the insulator effect of TAD boundaries, the increased hECR could not interact with *HMX1*, thus it probably does not change gene expression level.

Notably, the size of the hECR region (600 bp) is small and its copy number changes could be missed by chromosomal microarray analysis (CMA). Duplications detected in the present study range from ~ 50 to ~ 200 Kb. Although they were implicated in the Agilent SurePrint G3 Human CGH 1X1M microarray analysis, they could not be automatically detected in standard analysis process due to limited probes designed within the region. The genomic findings in these patients indicate the importance of checking the *HMX1*-ECR copy number status and highlight the necessity for custom designed microarrays with higher probe density covering this region.

## Conclusions

In this study, we found various genomic duplications involving the *HMX1*-ECR long range enhancer in five families with isolated bilateral concha-type Microtia. The *HMX1*-ECR duplications were specifically associated with isolated bilateral concha-type microtia but not with other ear malformations or syndromic microtia. We add to evidence in humans that copy number variations in *HMX1*-ECR, a conserved non-coding elements (CNEs), associates with ear malformations, as in other species. We provide additional evidence that the dosage sensitive effects of *HMX1* may result in different types of ear malformations. Unveiling genetic causes of isolated microtia provides an entry point into understanding the regulatory network for common lateral facial birth defects and complex syndromes involving external ear malformations. Meanwhile, the results could be used for genetic counseling and screening for isolated bilateral concha-type microtia.

## Supplementary information

**Additional file 1: Table S1.** Polymorphic micro-satellite markers and primers.

**Additional file 2: Table S2.** qPCR primers in 4p16.1.

**Additional file 3: Table S3.** Primers for plasmid construction.

**Additional file 4: Figure S1.** Visualization of chromatin interaction surrounding *HMX1*.

## Data Availability

All data generated or analyzed during this study were included in this published article and its additional files.

## Abbreviations

CNVs: Copy number variations
ECR: Evolutionarily conserved enhancer region
HMX1: The H6 family homeobox 1 transcription factor gene
SNP: Single nucleotide polymorphism
aCGH: Array comparative genomic hybridization
qPCR: Quantitative real-time polymerase chain reaction
OAS: Oculoauricular syndrome
SVs: Structural variants
WGS: Whole genome sequencing
hECR: Human sequence of evolutionarily conserved enhancer region
mECR: Mouse sequence of evolutionarily conserved enhancer region
DGV: Database of genomic variants
WHS: Wolf–Hirschhorn syndrome
OAVS: Oculoauriculovertebral spectrum
CNEs: Conserved non-coding elements
CRMs: Cis-regulatory modules
BA1 and BA2: First and second branchial arch
TADs: Topologically associated domains
CMA: Chromosomal microarray analysis
