# Supplementary material for: Duplications involving the long range HMX1 enhancer are associated with human isolated bilateral concha-type microtia
Source: J Transl Med. 2020 Jun 17;18:244. doi: 10.1186/s12967-020-02409-6 (PMC7302384; doi:10.1186/s12967-020-02409-6)
Supplement: Supplementary file 1 — Additional file 1: Table S1. Polymorphic micro-satellite markers and primers. [file 12967_2020_2409_MOESM1_ESM.docx]

Additional file 2: Table S1. Polymorphic micro-satellite markers and primers

| Markers | Primer Sequences | Cosegregation Status |
| --- | --- | --- |
| D4S3038 | Forward: GAAGACCAGCATTCGG | N |
|  | Reverse: GGTTTAATACACAGTAATTGTTCA |  |
| D4S3023 | Forward: ACCTCACTGGAAACTAAATGG | N |
|  | Reverse: TGAACAGCAGCGGTCT |  |
| CHLC.GATA151E03 | Forward: AAGATATTAGCAAAACAAATATGGC | Y |
|  | Reverse: CTTTAACTATCCTGACATTCCTAGG |  |
| D4S3352 | Forward: CTCCAGAGAAATAGAACCAATAAGA | N |
|  | Reverse: TAAGACTGAAACCAATTGGA |  |
| D4S2906 | Forward: CAGTCTAGATTCAAAGGAATTAGAC | N |
|  | Reverse: AATTAGAGATGCCCGTGAAA |  |
| D4S2998 | Forward: AAGTTCTTGGGCCGCAG | N |
|  | Reverse: TTCTACACCCAGGGGAACC |  |
| CHLC.GATA69C04 | Forward: AAGTTTGGAATTCAATATTGTGG | N |
|  | Reverse: GGGTTTATGGGAGTTATGAGCC |  |
| D4S2976 | Forward: TTCCACCCCCAAAAGA | N |
|  | Reverse: CAAAGGTTTTTAACATCCCA |  |
| D4S1566 | Forward: CATCCAAAATTAAAAAGTGC | N |
|  | Reverse: AATGTCAGGCCAGTTATGT |  |
| D4S1646 | Forward: CTCACCTAATAAGGCCCCTC | N |
|  | Reverse: AATGTACCACTTTGTCTTGAAGA |  |
| D5S504 | Forward: CCCAGAGGTACTTTATTGGATG | N |
|  | Reverse: ATTTGTAAGTTTTAAGGCTCCCC |  |
| D5S1961 | Forward: CCCATGAATGATTTGTCTAATGC | N |
|  | Reverse: ATGCCCTTTGCTGTCCA |  |
| D5S429 | Forward: CCCATGAATGATTTGTCTAATGC | N |
|  | Reverse: AATGCCCTTTGCTGTCCA |  |

N, not co-segregated with phenotype in F1; Y, co-segregated with phenotype in F1
