# Supplementary material for: Duplications involving the long range HMX1 enhancer are associated with human isolated bilateral concha-type microtia
Source: J Transl Med. 2020 Jun 17;18:244. doi: 10.1186/s12967-020-02409-6 (PMC7302384; doi:10.1186/s12967-020-02409-6)
Supplement: Supplementary file 2 — Additional file 2: Table S2. qPCR primers in 4p16.1. [file 12967_2020_2409_MOESM2_ESM.docx]

Additional file 3:Table S2. qPCR primers in 4p16.1

| Name | Sequences（5’-3’） | Amplicon Position |
| --- | --- | --- |
| q1 | Forward: GGCCGTCCCATTTTATAGAT | chr4:8617326-8617461 |
|  | Reverse: CAGGCCATGTCACTCTCTGA |  |
| q2 | Forward: GCTCCTGGCTCTTGATTTTG | chr4:8621401-8621488 |
|  | Reverse: TTCCAGTGCAAAGTGGATCA |  |
| q3 | Forward: AAAAGTCTGGGCTGTGTGGT | chr4:8639314-8639419 |
|  | Reverse: ACTGTGTGGAGGAGGACACC |  |
| q4 | Forward: GGCTCTGTGGAAGAGACCAG | chr4:8640744-8640844 |
|  | Reverse: CTCTCTGCAAATCCCCTCAC |  |
| q5 | Forward: CTGCATGGTTTTCACTCTGG | chr4:8666598-8666678 |
|  | Reverse: ATTGGCAGCAGCCGTATATC |  |
| q6 | Forward: CCCCAGTTCTCCTCTTACCA | chr4:8674850-8674968 |
|  | Reverse: CAGGGATGCAGTCAGGAGTC |  |
| q7 | Forward: ACAGCAAGAACCAGGAAGGA | chr4:8677186-8677318 |
|  | Reverse: TTGGCTGAGAAGCAGACTGA |  |
| q8 | Forward: TGCCAATCATGTCCCTCATA | chr4:8678265-8678381 |
|  | Reverse: TTCCCAGGAACAGACAATGA |  |
| q9 | Forward: TGGCATTTGGGGAGATTAGA | chr4:8681042-8681142 |
|  | Reverse: TGGATGGTAAAATCCTGCAA |  |
| q10 | Forward: TAGAGGAATCCCCCATTTCC | chr4:8682332-8682441 |
|  | Reverse: TGCTTTGCAGTGCAGAAATC |  |
| q11 | Forward: CTTTGCTTAGGCCCTGACTG | chr4:8682941-8683068 |
|  | Reverse: AAGGAACGAAGCTGCGATAA |  |
| q12 | Forward: AACAAGGATGCTCCCAGATG | chr4:8684896-8684995 |
|  | Reverse: ACCCAGGCTCTGCACATTAC |  |
| q13 | Forward: TGACCTTCCAGCAGAGGACT | chr4:8687696-8687839 |
|  | Reverse: CCCTTCTGCTGTTCCCAATA |  |
| **q14 /ECR** | **Forward: TTCCAACCCAGCGAAATTAG** | **chr4:8702020-8702157** |
|  | **Reverse: GGGATCAGTGGGAAGGAAGT** |  |
| q15 | Forward: GACTGGGAGGAGGCTTTCTT | chr4:8704275-8704356 |
|  | Reverse: GAAAACACTTCCCGTCCAGA |  |
| q16 | Forward: CTGGCCCTCTGTTCATTTGT | chr4:8705890-8706039 |
|  | Reverse: CCTCTTCTGACCCTGACACC |  |
| q17 | Forward: CCAAAAGCTTGAACCAAAGC | chr4:8706719-8706801 |
|  | Reverse: CTGAGGCACAAGACCTCACA |  |
| q18 | Forward: GTCGGGGAACTGGATAAGGT | chr4:8725480-8725577 |
|  | Reverse: ACAAGATCGGCTTCAGCACT |  |
| q19 | Forward: GACTGCAGCAGCATGAGAAG | chr4:8731972-8732105 |
|  | Reverse: ATGTGTGAAGGTGCCAGATG |  |
| q20 | Forward: GCCAGCCCTTGTAAGTCATC | chr4:8732509-8732632 |
|  | Reverse: GTCCCACAGGAACCTTCTCC |  |
| q21 | Forward: TCTCTCCCTCCTCTTGGTCA | chr4:8733848-8733943 |
|  | Reverse: TCAGCTTTTGCCTGGATTCT |  |
| q22 | Forward: CAGCCTGTCCACAGGAGAAG | chr4:8734548-8734695 |
|  | Reverse: ATGGGGTCCTAGAGGCAGAG |  |
| q23 | Forward: ACCACTCCGATGGTGATGTT | chr4:8736640-8736750 |
|  | Reverse: ATACTAGGCCCTGGGGAGAA |  |
| q24 | Forward: ACCTTGCTGCTCATTTGCTT | chr4:8738132-8738217 |
|  | Reverse: CAATATGGAAGAGCCCTGGA |  |
| q25 | Forward: ATGGGGTTGCTTCCTAAGGT | chr4:8740902-8740997 |
|  | Reverse: AAGATGTTGGGGATGACAGC |  |
| q26 | Forward: CGGATCAGAATCTTTGACCTC | chr4:8746164-8746285 |
|  | Reverse: AGTGCAGTGAGCATGACAGG |  |
| q27 | Forward: CCAACCCACGTTTTCAAGAT | chr4:8747843-8747959 |
|  | Reverse: CTGCTACACAGGGCATCAGA |  |
| q28 | Forward: CCCCAAGTTTTGTCCACTGT | chr4:8750406-8750554 |
|  | Reverse: GCAATGCGTTTTCACTGATG |  |
| q29 | Forward: TGCCAGAGAAGATGCATGAC | chr4:8762628-8762770 |
|  | Reverse: GGATGTGGCCACCAACTACT |  |
| q30 | Forward: CGTCAAGAGCCATGTCACAC | chr4:8774441-8774562 |
|  | Reverse: GCTGCTCCTAGGTGGATGAG |  |
| q31 | Forward: CCAGAGACTGCACTGATGGA | chr4:8785117-8785241 |
|  | Reverse: GGGGTTGATGGATTTCAGTG |  |
| q32 | Forward: CAGTCCAGACCCCAGACCTA | chr4:8796876-8797001 |
|  | Reverse: GCCCTGCTGATGGTGATAAT |  |
| q33 | Forward: AGCTCCTCCACCTTCTCCTC | chr4:8798083-8798174 |
|  | Reverse: ATTGCAGCATGCACACTTTC |  |
| q34 | Forward: CCAGGCTAGCTCTGCTTGTT | chr4:8819386-8819516 |
|  | Reverse: ACACCGTAGGGCTGTCATTC |  |
| q35 | Forward: GGGGTTGCAGATGTGAGAAT | chr4:8823313-8823441 |
|  | Reverse: TGAACACCATGGAACAGCAT |  |
| q36 | Forward: TGGGAGTGGAGTTTGTGAAC | chr4:8825739-8825832 |
|  | Reverse: CCTCCCCTGCACTGTTACAT |  |
| q37 | Forward: AGCAGCAGGCATTCTCATCT | chr4:8832332-8832435 |
|  | Reverse: AAGCTGCAGGGACAAGAAAA |  |
| q38 | Forward: TCTTCCAAGCCCTGATTAGC | chr4:8834414-8834547 |
|  | Reverse: CAGGGAGCCACATGAGAAAT |  |
| q39 | Forward: GGTTCTGGGTCACTCCCTTT | chr4:8839703-8839848 |
|  | Reverse: CCATGTTGCTTCTTGCACAT |  |
| q40 | Forward: GCCCAGGTTGACAGGTTAGA | chr4:8871135-8871246 |
|  | Reverse: CGTGCAAATGCTGCAAATAC |  |

qPCR assay in consensus human ECR region is highlight in bold.
