# Supplementary material for: Duplications involving the long range HMX1 enhancer are associated with human isolated bilateral concha-type microtia
Source: J Transl Med. 2020 Jun 17;18:244. doi: 10.1186/s12967-020-02409-6 (PMC7302384; doi:10.1186/s12967-020-02409-6)
Supplement: Supplementary file 3 — Additional file 3: Table S3. Primers for plasmid construction. [file 12967_2020_2409_MOESM3_ESM.docx]

Additional file 4: Table S3. Primers for plasmid construction

| **Primer** | **Sequences（5’-3’）** | **Restriction Enzyme/**  **Homologs sequences** | **Amplicon Length** |
| --- | --- | --- | --- |
| m-ECR-F | CGACGCGTGAATCCTGGCCAGTCAGTGTA | *Mlu*I | 591 bp |
| m-ECR-R | GGAAGATCTGGCTTGGGGGTGGCAAACTG | *BglI*I |  |
| hECR-F | TGGCCTAACTGGCCGGAACGCCGGCTTTGTGGG | Homologs sequences | 616 bp |
| hECR-R | TCTAGTGTCTAAGCTTTATTGGGGGGAAGAACCCGAGC | Homologs sequences |  |
| HOXA2-F | CCCAAGCTTGCCACCATGAATTACGAATTTGAGCG | *Hind*III | 1131 bp |
| HOXA2-R | CGGGATCCTTAGTAATTCAGATGCTGCA | *BamH*I |  |
