# Supplementary material for: Duplications involving the long range HMX1 enhancer are associated with human isolated bilateral concha-type microtia
Source: J Transl Med. 2020 Jun 17;18:244. doi: 10.1186/s12967-020-02409-6 (PMC7302384; doi:10.1186/s12967-020-02409-6)

**Additional Figure Legends**

**Additional Figure S1. Visualization of chromatin interaction surrounding *HMX1*.**  Hi-C profile data from human embryonic stem cells revealed two different TADs including *CPZ* and *HMX1* separately.


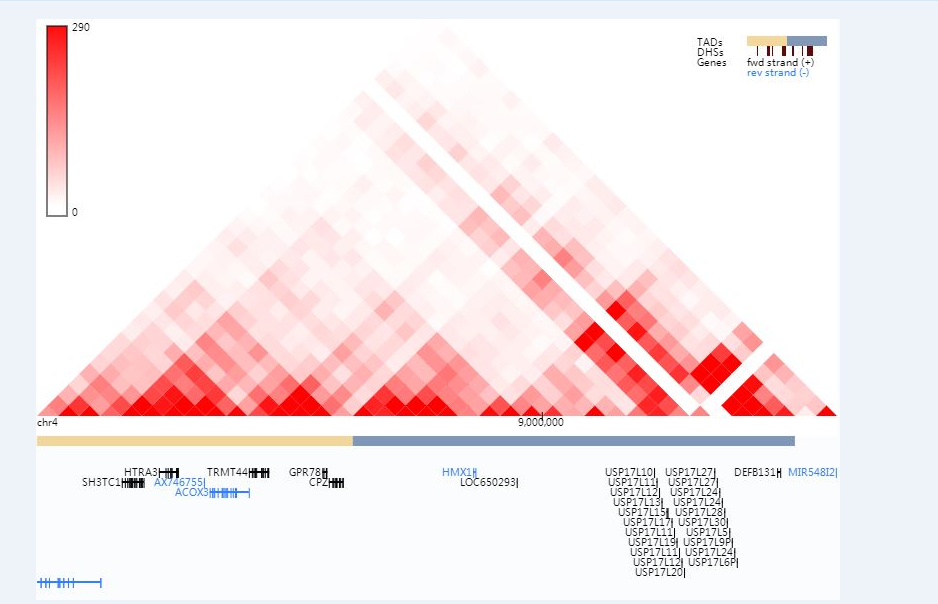

Supplement: Supplementary file 4 — Additional file 4: Figure S1. Visualization of chromatin interaction surrounding HMX1. [file 12967_2020_2409_MOESM4_ESM.docx]
